# Maryland Bridge: An Interim Prosthesis for Tooth Replacement in Adolescents

**DOI:** 10.5005/jp-journals-10005-1097

**Published:** 2010-04-15

**Authors:** P Prathyusha, Sapna Jyoti, Rupali Borkar Kaul, Ntasha Sethi

**Affiliations:** 1 Professor and Head, Department of Pedodontics and Preventive Dentistry, Dr Syamala Reddy Dental College Hospital and Research Center, Bengaluru, Karnataka, India; 2 Senior Lecturer, Department of Pedodontics and Preventive Dentistry, Dr Syamala Reddy Dental College Hospital and Research Center, Bengaluru, Karnataka, India; 3 Associate Professor, Department of Pedodontics and Preventive Dentistry, Dr Syamala Reddy Dental College Hospital and Research Center, Bengaluru, Karnataka, India; 4 Postgraduate Student, Department of Pedodontics and Preventive Dentistry, Dr Syamala Reddy Dental College Hospital and Research Center, Bengaluru, Karnataka, India

**Keywords:** Maryland Bridge, Interim prosthesis, Traumatized anterior tooth, Resin-bonded fixed partial denture.

## Abstract

A space in the anterior region of the dental arch of a youngster, either due to trauma or a congenitally missing tooth, can not only lead to psychological trauma but also create a functional dilemma for the dentist, as the usual treatment options of implant, removable partial denture and fixed partial denture available for adults, are often inapplicable or inconvenient for an adolescent. In such a situation, a resin-bonded fixed partial denture (RBFPD), such as Maryland Bridge fulfills all the requirements of an ideal interim solution till growth completion is achieved and a more permanent tooth replacement option can be explored.

## INTRODUCTION

Trauma to the anterior teeth is not uncommon, and one study reported that out of 2,100 children (aged 8-14 years) surveyed for teeth fractured due to trauma, 60.74% were aged between 11 and 14 with 13.8% cases involving incisors.^1^

With the significant advances dentistry has made, it is possible to save and restore such traumatized teeth using composites, crowns and post and core. But there are certain cases in which extraction is unavoidable, leaving us with an esthetic and functional dilemma for the adolescent patient. For such cases, a Maryland Bridge may prove to be an ideal option, as the case has been.

## CASE REPORT

**Fig. 1:**
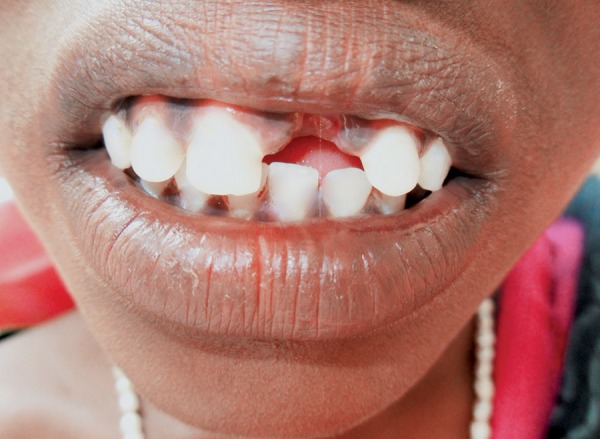
Patient reports with fractured maxillary left central incisor

**Fig. 2:**
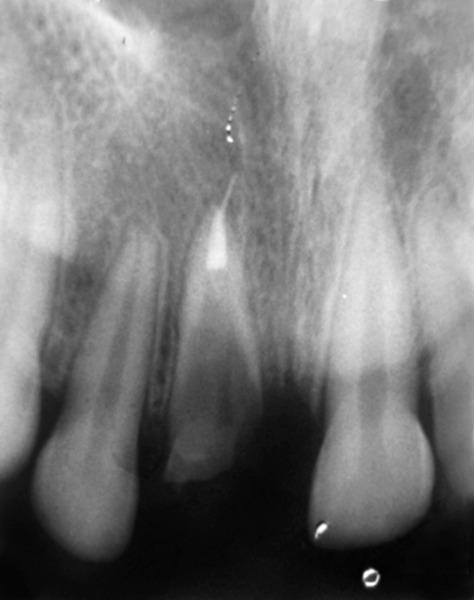
IOPAR showing root stump of the fractured maxillary left central incisor highlighting the internal and external resorption

A female patient, aged 13 years, presented with a fractured maxillary left central incisor and desired a stable esthetic solution (Fig. 1). Three years ago, patient had fractured the tooth due to a fall. Patient did not give any clear history about the dental treatment she received after the fracture. On examination, it was revealed that no crown structure was visible clinically and only a root stump remained in relation to the aforementioned tooth. Apart from that the patient had generalized staining and spacing between the maxillary anterior teeth. On radiographic examination, it was revealed that apical third of root canal was obturated with 4 mm of gutta-percha (Fig. 2). Periapically, external root resorption was noticed with 3 mm of gutta-percha extending beyond the apex, indicating a previous failed root canal treatment.

After considering the patient’s wishes and the clinical situation, the options of post and core, removable partial denture, fixed partial denture and implant were eliminated. Finally, it was decided to extract the remaining root stump and replace it with a Maryland Bridge as an interim solution.

**Fig. 3:**
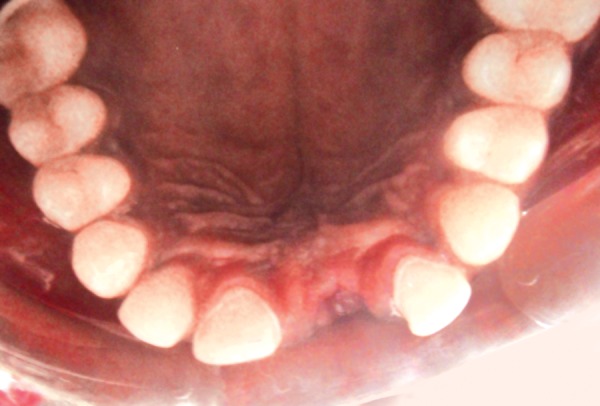
The tooth preparation of maxillary right central incisor and left lateral incisor on the palatal aspect

After completing oral prophylaxis, extraction of the root stump was done. Tooth preparation for both 11 and 22 was done following the standard technique.^2^ Lingual preparation ended 2 mm from the incisal edge and a light chamfer finish line was prepared 1 mm supragingivally (Fig. 3). An impression was made in polyether impression material and sent to the laboratory. After the metal try-in was successful, shade selection was done using a shade guide. The trial fitting of the prosthesis was done and then esthetics, mastication and speech were evaluated. In this case, the esthetics had to be compromised slightly as the edentulous space was wider than the mesiodistal width of the original tooth, leading to an oversized pontic. It was therefore elected to cover the metal retainers with porcelain.

**Fig. 4:**
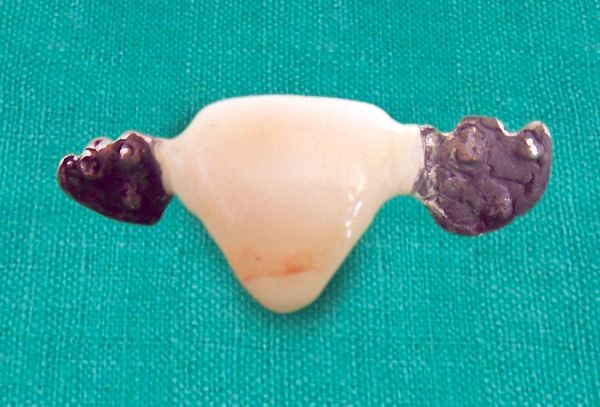
Preparation of webbings on the incisal edge of the metal wings

**Fig. 5:**
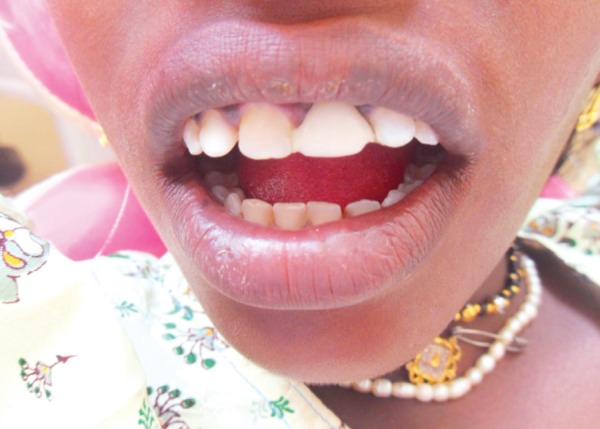
Postoperative picture of the patient after cementation of the Maryland Bridge (labial view)

**Fig. 6:**
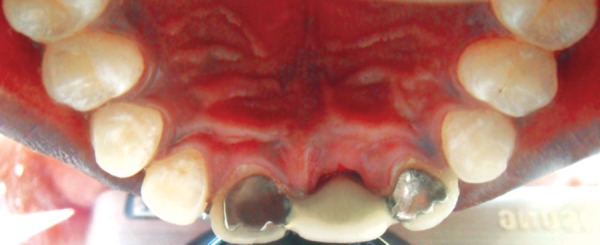
Postoperative picture of the patient after cementation of the Maryland Bridge (palatal view)

Before cementation, a modification was made in the wings of the Maryland Bridge by producing webbings on the incisal edge (Fig. 4) with coarse diamond bur which leaves a good roughened surface. This simple modification requires little time and provides increased retention.^3^ After isolation with a rubber dam, the Maryland bridge was cemented using a resin cement (Figs 5 and 6) followed by macro-bonding in the webbed area using a conventional composite resin. A 6-month follow-up was advised until the patient is ready to replace the bridge with a more permanent solution.

## DISCUSSION

Replacement of a missing or grossly decayed/fractured tooth requires a fine balancing by the dentist of the functional and psychological factors involved.

The first option for a severely fractured tooth is always root canal followed by post and core, but in this case the apical seal had already been compromised. Also, for teeth missing all of the coronal tooth structure to the level of the gingival tissue, the prognosis for post and core is questionable.^4^

Removable partial dentures are the cheapest and the most easily fabricated options but they are often unacceptable to the patient because they are bulky, uncomfortable and not very esthetically pleasing, often leading to papillary hyper-plasia if proper oral hygiene is not maintained.^5^

The next option that can be explored is a fixed partial denture which requires significant tooth reduction. The enlarged pulp chamber in an adolescent may prompt the clinician to make an underprepared tooth with a resulting oversized finished crown. Increased pulpal response during tooth preparation and later the possible exposure of the crown margins as natural apical migration of the epithelial attachment proceeds with age, may also act as deterrents.^6^ Further, the longevity of the fixed partial denture is reported to be 8.3 to 10.3 years, requiring replacement three or four times over the course of a young patient’s life with the additional loss of tooth structure.^7^

Among the most acceptable and conservative options available currently for replacement of missing teeth are implants. But continued bone growth in an adolescent may lead to infraocclusion of the implant relative to other teeth and in some cases periodontal problems may arise with marginal bone loss around the implant and adjacent teeth.^8^

With the introduction of the adhesive-retained fixed partial denture, by Livaditis in 1980,^2^ a new era of conservative tooth replacement has dawned.

A cantilever bridge would have been an ideal option in the current case, owing to the tooth-pontic size discrepancy, but for the replacement of a maxillary central incisor the lateral incisor did not offer adequate coverage area and the bridge could not be cantilevered across the midline by bonding to the central incisor.^9^

A Maryland Bridge offers multiple advantages, such as minimal tooth preparation, involving removal of less than half the amount of coronal tooth structure by weight compared to complete coverage crowns.^10^ An esthetically satisfactory result can be achieved at an affordable cost while minimizing the chair side time. Patient comfort is enhanced by the fact that anesthesia is avoided and the pulpal trauma is minimal. Other merits are easy impression making due to supragingival margins and avoidance of any interim restoration. Even after 10 years of service the periodontal response for resin-bonded fixed partial dentures is minimal and is comparable to periodontal response to other types of restorations.^11^

The three most common complications associated with resin-bonded prosthesis are debonding (21%), tooth discoloration (18%) and caries (7%).^12^ Overall survival rate has been computed as being 77% after 10 years of service.^13^ Conversely, it is also true that rebonding or reconstruction of the metal frame after dislodgement increased the survival rate to 87% after 8 years under risk.^14^ Excellent results are achieved in patients with small edentulous spans bounded by sound teeth, having an adequate crown height and width.^2^

A study involving 358 patients concluded that the degree of satisfaction with RBFPDs was high and did not seem to be influenced by the occurrence of failure.^15^

Careful case selection, judicious design planning, precise preparation and meticulous cementation regimen can all ensure the long-term success of Maryland Bridges, making them ideal candidates for temporary replacement of single anterior missing tooth in adolescents.
